# Oral Immunization with *Lactobacillus casei* Expressing the Porcine Circovirus Type 2 Cap and LTB Induces Mucosal and Systemic Antibody Responses in Mice

**DOI:** 10.3390/v13071302

**Published:** 2021-07-05

**Authors:** Fengsai Li, Xiaona Wang, Rumeng Ma, Wei Wu, Fei Teng, Xi Cheng, Yanping Jiang, Han Zhou, Li Wang, Lijie Tang, Xinyuan Qiao, Yijing Li

**Affiliations:** 1 College of Veterinary Medicine, Northeast Agricultural University, Harbin 150030, China; Yilvwenrou@126.com (F.L.); xiaonawang0319@163.com (X.W.); mrm0134@163.com (R.M.); ww13936206166@163.com (W.W.); teng1085579571@163.com (F.T.); 18008349515@163.com (X.C.); jiangyanping2017@126.com (Y.J.); zhouhan9659@163.com (H.Z.); wanglicau@163.com (L.W.); tanglijie@neau.edu.cn (L.T.); 2 Heilongjiang Key Laboratory for Animal Disease Control and Pharmaceutical Development, Harbin 150030, China

**Keywords:** *Lactobacillus casei*, PCV2, Cap, LTB, oral administration

## Abstract

Porcine circovirus type 2 (PCV2) causes many diseases in weaned piglets, leading to serious economic losses to the pig industry. This study investigated the immune response following oral administration of *Lactobacillus casei ATCC393* (*L. casei 393*) expressing PCV2 capsid protein (Cap) fusion with the *Escherichia* *coli* heat-labile toxin B subunit (LTB) in mice. Recombinant *L. casei* strains were constructed using plasmids pPG611.1 and pPG612.1. The expression and localization of proteins from recombinant pPG611.1-Cap-LTB (pPG-1-Cap-LTB)/*L. casei 393* and pPG612.1-Cap-LTB (pPG-2-Cap-LTB)/*L. casei 393* were detected. All recombinant strains were found to be immunogenic by oral administration in mice and developed mucosal and systemic immune responses against PCV2. The titers of specific antibodies in mice administered pPG-2-Cap-LTB/*L. casei 393* were higher than those in mice administered pPG-1-Cap-LTB/*L. casei 393* in serum and the mucosal samples. The mucosal immune response was not only limited to the gastrointestinal tract but was also generated in other mucosal parts. Thus, the application of recombinant *L. casei* could aid in vaccine development for PCV2.

## 1. Introduction

Porcine circovirus type 2 (PCV2) is etiologically associated with a complex of PCV diseases, including post-weaning multisystemic wasting syndrome (PMWS) [1]. PMWS primarily affects pigs from 5 to 12 weeks of age, which can develop severe immunosuppression with morbidities of 5–30%, resulting in substantial economic losses to the pig industry [2]. PCV2 is also associated with respiratory disease, reproductive failure, hepatitis, and porcine dermatitis and nephropathy syndrome [1,3]. PCV2 is a small, non-enveloped, single-stranded, circular DNA virus with a 1767 or 1768 nt ambisense genome [4] that contains at least two major open reading frames (ORFs). ORF1 encodes the replication proteins (Rep and Rep’) involved in virus replication, and ORF2 encodes the capsid protein (Cap) [5,6]. Cap, associated with the generation of neutralizing antibodies and the protection of antibodies [7,8], is a candidate target for the design of new vaccines against PCV2 infection.

The control of PCV2 is based on management strategies, the control of coinfections, and vaccination. The induction of an active immune response against the ORF2-encoded Cap is the major immunogenic mechanism to protect piglets from PCV2 attack [9,10]. To stimulate an effective immune response, a large vaccine dose and repeated administration is usually required, which can result in some undesirable clinical symptoms. To overcome these shortcomings, the potential development of lactic acid bacteria (LAB) to deliver heterologous antigens to the mucosal immune system has been proposed.

Compared to recombinant antigens or heat-killed formulations, ‘live’ vaccines elicit the most effective protective responses because they stimulate both systemic and mucosal immunity [11,12,13,14]. However, oral vaccination presents a challenge because the gut milieu denatures and/or inactivates potential vaccinogens [15,16]. This often results in fecal shedding of the live vaccine, in addition to causing fever and diarrhea [16,17,18]. These challenges could be overcome by using LAB as an antigen delivery system for the stimulation of mucosal immunity [17,18,19,20,21,22] owing to its safety. In addition, many strains of LAB can survive and colonize the intestinal tract [23,24], inducing a non-specific immunoadjuvant effect [25], which has prompted the investigation of the oral vaccine potential of LAB-derived vaccines.

In this study, we tested the efficacy of *Lactobacillus casei ATCC 393* (*L. casei 393*) acting as an antigen delivery system to express the heterologous PCV2 Cap protein for oral vaccinations. The *Escherichia coli* heat-labile toxin B subunit (LTB) was combined as an adjuvant to increase immunogenicity, which is a potent mucosal adjuvant [26,27,28,29,30] with low potential to elicit allergic responses [27,31]. We constructed two recombinant strains expressing Cap-LTB with the immunogen analyzed by the oral administration of live bacteria to mice. The results indicated that the oral administration of two recombinant strains, pPG611.1-Cap-LTB (pPG-1-Cap-LTB)/*L. casei 393* and pPG612.1-Cap-LTB (pPG-2-Cap-LTB)/*L. casei 393*, could induce specific anti-PCV2 mucosal and systemic immune responses.

## 2. Materials and Methods

### 2.1. Bacterial Strain and Culture Conditions

*L. casei 393* was kindly supplied by Prof. Jos Seegers (NIZO, Ede, The Netherlands) and cultured in sterile Man, Rogosa, and Sharpe (MRS) broth at 37 °C anaerobically without shaking. To analyze the expression of Cap-LTB protein, recombinant strains were grown in basal MRS medium supplemented with 2% xylose. The antibiotic concentration used for the selection of transformants was 10 μg/mL of chloromycetin (Sigma, Ronkonkoma, NY, USA) if necessary. The plasmids pPG611.1 and pPG611.2 were kindly gifted by Prof. Jos Seegers (NIZO, Ede, The Netherlands).

### 2.2. Animals

BALB/c clean mice weighing 25–30 g (seven weeks of age) were obtained from the Harbin Veterinary Research Institute. The experimental and control group for each period of the assay consisted of 10 mice.

### 2.3. Plasmid Procedure and Electrotransformation

**Figure 1:**
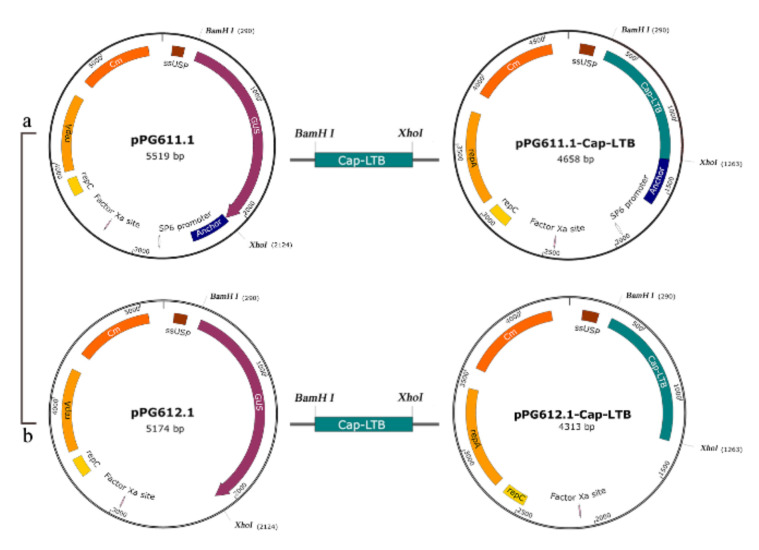
Schematic diagram of the construction of DNA plasmids. (**a**,**b**) The genes Cap and LTB were amplified with the plasmid pMD18-Ts-Cap/LTB fused by polymerase chain reaction (PCR) and then inserted into the vectors pPG611.1 or pPG612.1 at *BamH* I and *Xho* I sites, generating plasmids pPG611.1-Cap-LTB and pPG612.1-Cap-LTB.

A schematic diagram for the construction of recombinant plasmids is shown in Figure 1. The fragment, approximately 576 bp, encoding the Cap gene of PCV2 was amplified from the plasmid pMD18-Ts-Cap by polymerase chain reaction (PCR) with the primers C0/C1 (Table 1). The gene encoding LTB was obtained from the plasmid pMD18-Ts-LTB (L1/L2), then fused to the 3′ terminus of the Cap gene by fusion PCR with the complementary sequence of the primers C1 and L1. A flexible short peptide GPGPLV was introduced at the splicing of the two genes. The PCR product of the Cap-LTB gene was generated and inserted into the corresponding sites of pPG611.1 and pPG612.1 at *BamH* I and *Xho* I restriction endonuclease sites, giving rise to the recombinant plasmids pPG-1-Cap-LTB and pPG-2-Cap-LTB, respectively [29]. The two plasmids both allowed the Cap protein to be expressed outside the bacteria without further being destroyed.

Electrotransformation of *L. casei 393* was carried out by adding recombinant plasmid DNA (1 μg) to 200 μL of *L. casei 393* competent cells, gently mixing at 4 °C for 5 min, and subjecting them to a single electric pulse (25 μF of 2.5 kV/cm). The mixture was incubated in MRS medium without chloromycetin (Cm) at 37 °C anaerobically for 4 h. Recombinant strains were selected on MRS agar medium containing 10 μg/mL of Cm. The presence and integrity of the constructions carried by the *L. casei 393* transformants were checked by the extraction of recombinant plasmid DNA before sequencing.

### 2.4. Protein Expression and Western Blotting Analysis

To analyze the expression of Cap-LTB by xylose-induced pPG-1-Cap-LTB/*L. casei 393* and pPG-2-Cap-LTB/*L. casei 393* overnight, cultures grown in basal MRS broth supplemented with xylose were collected by centrifugation at 12,000× *g* for 10 min. The pellets were washed twice with sterile 50 mM Tris-Cl at pH 8.0 and treated with 10 mg/mL lysozyme at 37 °C for 60 min. The lysates were centrifuged at 12,000× *g* for 10 min and applied to the protein analysis.

Protein extractions were subjected to 15% sodium dodecyl sulfate–polyacrylamide gel electrophoresis (SDS-PAGE) and Western blot assay. The proteins were separated by SDS-PAGE and then electrotransferred on a nitrocellulose membrane, and the blots were developed using rabbit anti-Cap (polyclonal antibody prepared by purified PCV2) serum (previously prepared in our laboratory) at a dilution of 1:500 with phosphate-buffered saline (PBS) (pH 7.4). Horseradish peroxidase (HRP)-conjugated goat anti-rabbit IgG (Sigma) was utilized as a secondary antibody at a dilution of 1:8000, which was visualized with the Chemiluminescent Substrate reagent (Pierce, Rockford, IL, USA) following the manufacturer’s instruction. The recombinant *L. casei* harbored empty plasmids pPG611.1 and pPG611.2, which acted as negative controls, named pPG611.1 (pPG-1)/*L. casei 393* and pPG611.2 (pPG-2)/*L. casei 393*, respectively.

### 2.5. Surface Expression Analysis

Immunofluorescence was used to analyze protein expression by pPG-1-Cap-LTB/*L. casei 393*, as described previously [32]. Briefly, 2 mL of induced cultures was harvested to an OD_600_ of 0.5–0.6, resuspended in 1 mL of sterile PBS and 3% bovine serum albumin (BSA) containing anti-Cap antibodies, and then incubated overnight at 37 °C. The cell precipitate was washed three times with sterile PBS 0.05% Tween 20. The cell–antibody complexes were then incubated for 6 h at 37 °C in the dark with fluorescein isothiocyanate (FITC)-conjugated goat antirabbit IgG (Sigma, Ronkonkoma, NY, USA). Cells were washed three times with PBS 0.05% Tween 20 and then air-dried on a glass slide. The surface expression analysis was performed using a confocal microscope. Non-induced recombinant strains were used as negative controls.

### 2.6. Secretion Expression Analysis

The supernatant of pPG-2-Cap-LTB/*L. casei 393* was separated by SDS-PAGE and electrotransferred on a nitrocellulose membrane and the blots were detected using rabbit anti-Capserum at a dilution of 1:500 with PBS (pH 7.4). HRP-conjugated goat anti-rabbit IgG (Sigma) was utilized as a secondary antibody at a dilution of 1:8000, which was visualized with the Chemiluminescent Substrate reagent (Pierce, Rockford, IL, USA) following the manufacturer’s instructions. The supernatant of the non-induced recombinant strain was used as the negative control.

Furthermore, to analyze whether the protein of interest was secreted to the supernatant of the recombinant strains pPG-2-Cap-LTB/*L. casei 393*, an indirect enzyme-linked immunosorbent assay (ELISA) was performed according to a previously described method [33]. The supernatant of recombinant bacteria pPG-2-Cap-LTB/*L.* *casei 393* was concentrated by trichloroacetic acid by 50-fold, and coated in a 96-well ELISA reaction plate to analyze the target protein [34]. The supernatant of pPG-2/*L. casei 393* was used as a negative antigen control, and rabbit negative serum was used as an antibody control. Polystyrene microtiter plates were coated overnight at 4 °C. After the wells were blocked for 2 h at 37 °C with PBS containing 5% skim milk, rabbit anti-Cap serum was used as the primary antibody diluted in PBS containing 5% skim milk and was incubated for 1 h at 37 °C. The plates were washed three times with 0.05% PBS Tween 20, and HRP-conjugated goat anti-rabbit IgG antibody (Invitrogen, Carlsbad, CA, USA) was added into each well (1:5000) and incubated for an additional 1 h at 37 °C. Another round of washing and color development was carried out using o-phenylenediamine dihydrochloride as a substrate, and absorbance was measured at 490 nm. Differences in the samples between treatments were analyzed for the level of significance by ANOVA.

### 2.7. Immunizations

pPG-1-Cap-LTB/*L. casei 393* and pPG-2-Cap-LTB/*L. casei 393* were cultured and centrifuged as described above. Cell pellets were washed once with sterile PBS and resuspended in PBS (pH 7.4). Mice were orally vaccinated with 0.2 mL of 10^9^ colony-forming units (c.f.u.)/mL of the recombinant strains. A control group of 10 mice received PBS. Mice in all groups were immunized on three consecutive days at days 0–2, 14–16 (first booster), 28–30 (second booster), and 42–44 (third booster).

### 2.8. Enzyme-Linked Immunosorbent Assay

Mouse serum, feces, and vaginal washes were collected on days 7, 21, 35, and 49 to detect the specific anti-Cap antibodies by ELISA. Intestinal mucus was collected on days 0 and 49 as described previously [35]. Fecal samples, vaginal washes, and intestinal mucus were collected to detect the SIgA antibody and treated according to methods described previously, with slight modifications [36]. In brief, 0.1 g of fecal pellets was sampled and subsequently suspended in 500 μL of PBS containing 50 mmol/L EDTA-Na2; after incubating at 4 °C for 16 h and centrifuging at 12,000 rpm for 2 min, the supernatants were acquired. The supernatant was collected, after scraping the small intestinal mucus with 500 μL PBS and mixed well. Vaginal washes were gained by rinsing the vaginas of mice repeatedly. Serum was applied for IgG antibody detection with tenfold dilution. All samples were stored at −20 °C until they were subjected to ELISA.

Polystyrene microtiter plates were coated overnight at 4 °C with PCV2 propagated on PK15 cells or supernatants harvested from PK15 cells cultured without PCV2 as the negative control. After the wells were blocked for 2 h at 37 °C, mucosal or serum samples were serially diluted in PBS-1% BSA and added in triplicates, incubating for 1 h at 37 °C. After the plates were washed three times with 0.05% PBS Tween 20, HRP-conjugated goat anti mouse IgG or IgA antibody (Invitrogen) was added to each well (1:5000) and incubated for an additional 1 h at 37 °C. After another round of washing, color development was carried out using o-phenylenediamine dihydrochloride as the substrate, and then absorbance was measured at 490 nm. Differences in the samples between treatments were analyzed for the level of significance by ANOVA.

### 2.9. Lymphocyte Proliferation

On day 40 after primary immunization, three mice from each group were euthanized to prepare splenocytes under aseptic conditions for conducting the lymphocyte proliferation assay, as previously described [37]. In brief, after the mice were euthanized, a lymphocyte suspension was prepared by adjusting the number of cells to 5 × 10^6^/mL, cultured in RPMI-1640, and containing 20% fetal calf serum at 37 °C with 5% CO_2_ in a 96-well plate. The cells were stimulated with 0.5, 2.5, 25, and 50 μg/mL of purified PVC2 Cap recombinant protein (specific antigen stimulation) for 66 h at 37 °C. In parallel, stimulation with 5 μg/mL of concanavalin A (ConA) and RPMI-1640 was used as the positive and negative control, respectively. With absorbance measured at 490 nm, lymphocyte proliferation was evaluated using the Cell Titer 96 Aqueous Non-Radioactive Cell Proliferation Assay (Promega, Madison, WI, USA) according to the manufacturer’s instructions.

### 2.10. Statistical Analysis

Statistical significance was determined using ANOVA, with a *p*-value < 0.05 considered as significant.

## 3. Results

### 3.1. Expression of Cap-LTB in L. casei

The sequences of *L. casei 393* transformants were confirmed by plasmid DNA sequencing, and the results showed that there were no mutations in the transformants.

**Figure 2:**
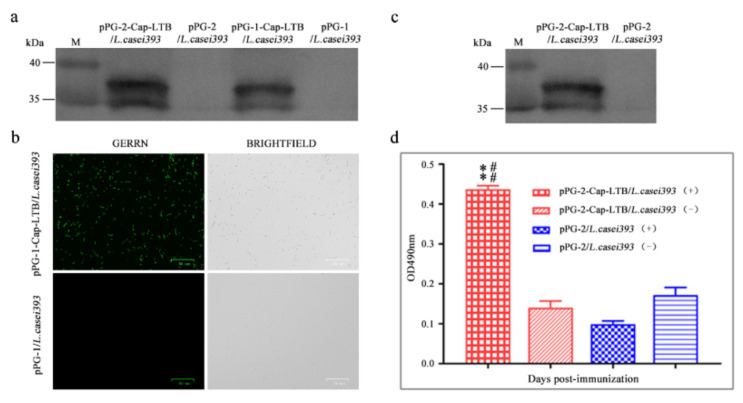
Analysis of the expression of the protein of interest of recombinant *L. casei 393* strains. (**a**,**b**) Fusion protein expression on the surface of the cells was analyzed via Western blotting and immunofluorescence using rabbit anti-Cap serum; (**a**) The results show a relevant immunoreactive band in pPG-1-Cap-LTB/*L. casei 393* and pPG-2-Cap-LTB/*L. casei 393*, but none in the empty cells. M: protein molecular weight markers; (**b**) There was a green fluorescent signal on the cell surface of strain pPG-1-Cap-LTB/*L. casei 393*, but not of strain pPG-1/*L. casei 393*, indicating that the protein of interest was expressed and displayed successfully on the surface of recombinant LAB; (**c**,**d**) Fusion protein secretory expression at the supernatant of the cells was analyzed via Western blotting and indirect ELISA using rabbit anti-Cap serum. Bars represent the mean ± standard error value of each group (* *p* < 0.05, ** *p* < 0.01 compared to the control groups: pPG-1/*L. casei 393* and # *p* < 0.05, ## *p* < 0.01 compared to group: pPG-2/*L. casei 393*).

pPG-1-Cap-LTB/*L. casei 393* and pPG-2-Cap-LTB/*L. casei 393* were grown overnight in basal MRS medium supplemented with xylose. The lysates of the cells were analyzed by Western blotting. Protein extractions were transferred onto nitrocellulose membranes and detected using anti-Cap antibodies. Immunoreactive bands corresponding to Cap-LTB showed that the fusion protein was expressed in pPG-1-Cap-LTB/*L. casei 393* and pPG-2-Cap-LTB/*L. casei 393* induced by xylose at approximately 35 and 37 kDa (Figure 2a, lane 3 and Figure 2a, lane 1), but not in basal MRS medium with the empty cells (Figure 2a, lane 4 and Figure 2a, lane 2). These results demonstrate the efficiency of the *L. casei* xylose promoter; the fusion protein was expressed successfully in recombinant strains.

### 3.2. Cap-LTB Protein Surface-Displayed Expression by L. casei

The pellet of overnight cultures of pPG-1-Cap-LTB/*L. casei 393* induced in basal MRS supplemented with xylose was collected and analyzed by immunofluorescence. The immunofluorescence reaction of pPG-1-Cap-LTB induced by xylose developed with the rabbit anti-Cap serum and FITC-conjugated goat anti-rabbit IgG also showed that there was green fluorescence only on the cell surface of pPG-1-Cap-LTB/*L. casei 393* (Figure 2b) and not on that of the non-induced recombinant strains (Figure 2b). These results indicated that the fusion protein Cap-LTB was successfully expressed on the surface of the bacteria.

### 3.3. Cap-LTB Protein Secretory Expression by L. casei

Supernatant of the overnight culture of pPG-2-Cap-LTB/*L. casei 393* in basal MRS medium with additional xylose was obtained by centrifugation, which was subjected to immunoblotting to detect the target protein expression. An immunoreactive band of 37 kDa was detected in the supernatant (Figure 2c, lane 1), whereas there was no band in the negative control lane (Figure 2c, lane 2). The results demonstrated that expressed Cap-LTB protein was secreted into the supernatant after induction by xylose in *L. casei* 393.

The target protein in the supernatant was also detected by indirect ELISA with the recombinant bacteria pPG-2-Cap-LTB/*L. casei 393*, rabbit anti-Cap serum as the primary antibody, and HRP-labeled goat anti-rabbit IgG as the secondary antibody. The results showed that the OD value of the recombinant bacteria and positive sera reaction was significantly higher than that of other bacteria and positive sera, although the numerical value of the control group and the recombinant bacteria, with the reaction of negative serum was not obvious, indicating that Cap-LTB protein was secreted into the supernatant in *L. casei 393*.

### 3.4. Antibody Responses Following Oral Immunizations

**Figure 3:**
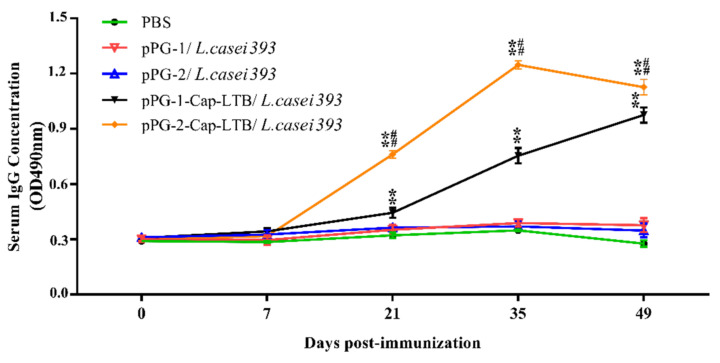
Specific IgG antibody levels in mice post-immunization. Measurement of specific anti-PCV2 IgG by an ELISA using PCV2 as the coating antigen. Bars represent the mean SE in each group (* *p* < 0.05, ** *p* < 0.01 compared to the control groups: pPG-1/*L. casei 393* and # *p* < 0.05, ## *p* < 0.01 compared to group: pPG-2/*L. casei 393*).

The ability of the Cap-LTB-expressing *L. casei* vectors to elicit systemic and/or mucosal immunity was assessed by determining the presence of specific IgG and/or IgA antibodies, respectively. IgG antibody levels in the serum of mice treated with pPG-2-Cap-LTB/*L. casei 393* were higher than those in mice treated with pPG-1-Cap-LTB/*L. casei 393*, both of which were higher than those in mice treated with PBS only (Figure 3). After the first booster, a prompter and stronger level of specific serum IgG was elicited in mice that were administered recombinant strains. A statistically significant difference was observed on day 21, 35, and 49 (** *p* < 0.01, Figure 3). No significant elicitation of specific antibodies was observed in the control group that received PBS.

**Figure 4:**
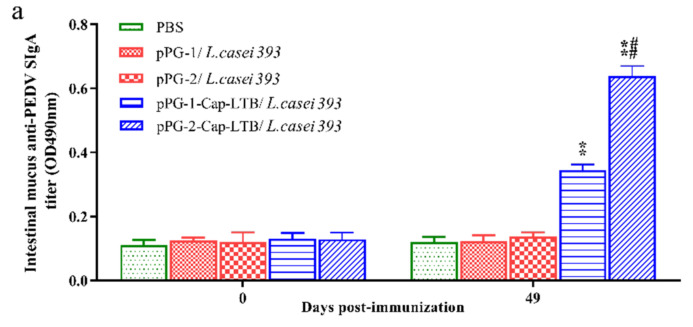

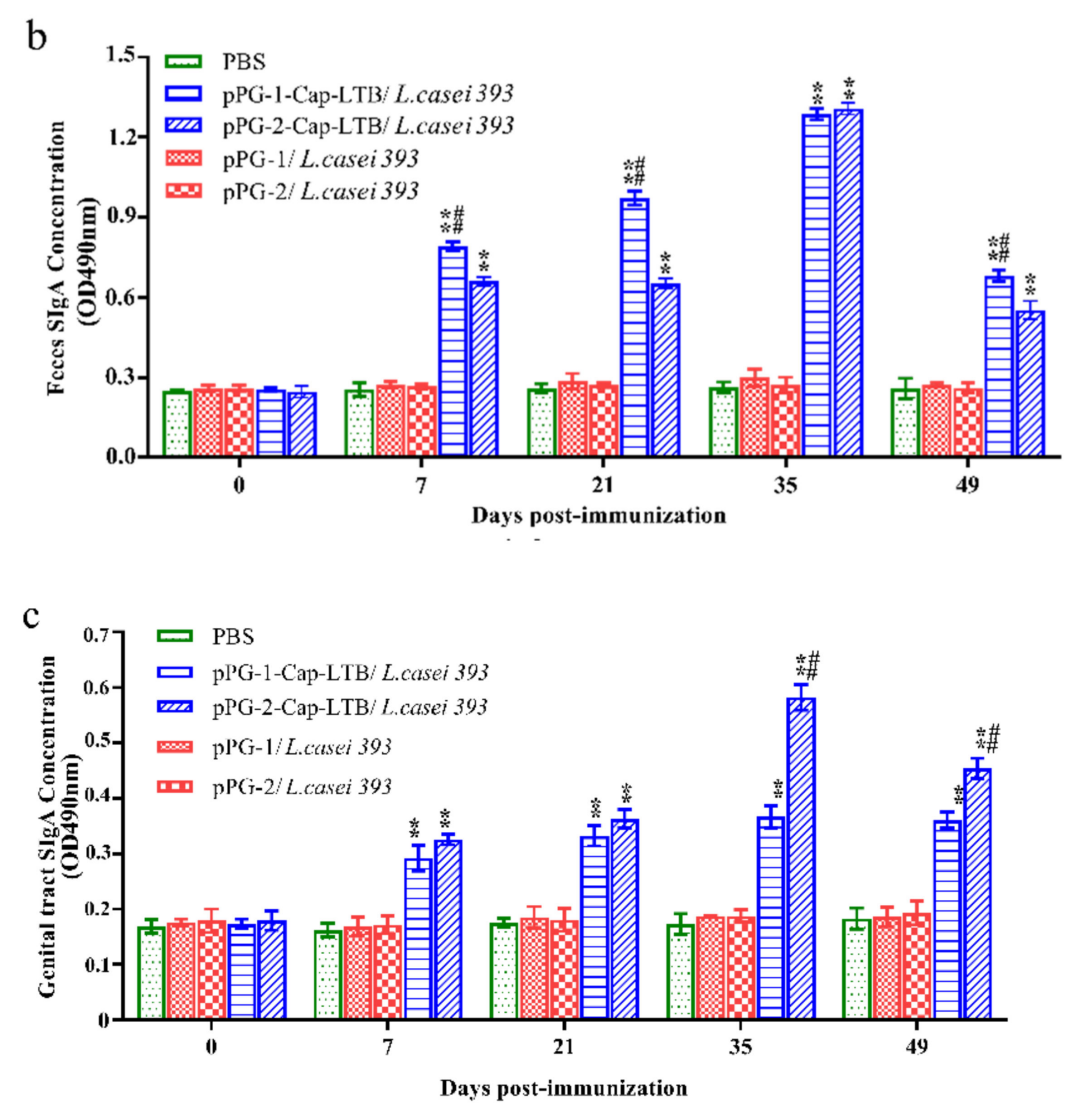
Specific IgA antibody levels in mice post-immunization. Determination of anti-PEDV mucosal SIgA antibody in (**a**) feces, (**b**) intestinal mucus, and (**c**) vaginal wash by ELISA using PCV2 as the coating antigen. Bars represent the mean ± standard error of each group (* *p* < 0.05, ** *p* < 0.01 compared to the control groups: pPG-1/*L. casei 393* and # *p* < 0.05, ## *p* < 0.01 compared to group: pPG-2/*L. casei 393*).

Seven days after the first immunization, higher levels of IgA-specific antibodies were observed in the mice treated with pPG-1-Cap-LTB/*L. casei 393* or pPG-2-Cap-LTB/*L. casei 393* in feces and vaginal wash (Figure 4b,c), which were significantly different from the control mice that received PBS (** *p* < 0.01). Similar results were shown in intestinal mucus at 49 days with immunization (Figure 4a). The mucosal IgA levels elicited by pPG-2-Cap-LTB/*L. casei 393* were higher than those elicited by pPG-1-Cap-LTB/*L. casei 393* immunization. These results showed that the mice immunized with recombinant bacteria induced the production of systemic and mucosal immunity, and the immune effect of the secreted recombinant *L. casei* was better than that expressed on the cell surface.

### 3.5. Lymphocyte Proliferation

**Figure 5:**
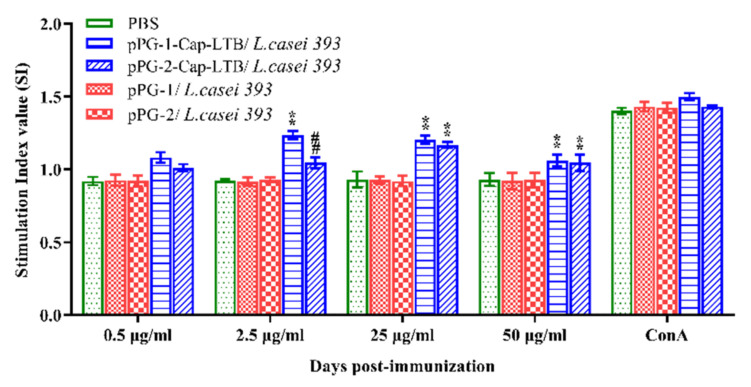
Lymphocyte proliferation in immunized mice detected by 3-(4,5-dimethylthiazol-2-yl)-2,5-diphenyltetrazolium bromide (MTT) assay in response to PCV2 Cap protein as a stimulating agent. Bars represent the mean ± standard error of each group (* *p* < 0.05, ** *p* < 0.01 compared to the control groups: pPG-1/*L. casei 393* and # *p* < 0.05, ## *p* < 0.01 compared to group: pPG-2/*L. casei 393*).

Lymphocyte proliferation splenocytes were isolated from immunized mice and restimulated with the PCV2 Cap recombinant protein in vitro to detect the lymphocyte proliferation response by 3-(4,5-dimethylthiazol-2-yl)-2,5-diphenyltetrazolium bromide (MTT) assay using ConA as the positive control and cell culture medium as the negative control. As shown in Figure 5, the stimulation index significantly increased in the mice immunized with pPG-2-Cap-LTB/*L. casei 393* and pPG-1-Cap-LTB/*L. casei 393* compared to those immunized with PBS, and there were no obvious changes in the groups with pPG-1/*L. casei 393* and pPG-1/*L. casei 393* compared to the PBS group, indicating that the mice immunized with recombinant bacteria could induce the production of systemic immunity.

## 4. Discussion

PCV-associated disease, which is caused by PCV2, is one of the most economically important diseases in the global swine industry [38,39]. Vaccination is a key tool for the control of PCV2 infection. Due to the inherent particularity of the porcine circovirus, there are some features, such as no pathological changes or low virus titer on the cells, which bring certain difficulties to the development of traditional fire-fighting vaccines and attenuated vaccines [40]. Therefore, the development of new vaccines has attracted considerable attention [41,42,43]. ORF2 is the second largest ORF of PCV2, encoding the Cap protein of the virus, which is also the main structural protein of the virus that induces the body to produce neutralizing antibodies [41]. Porntippa et al. used the baculovirus expression system to achieve high-efficiency expression of the PCV2-ORF2 gene in insect cells. With the protein being purified, virus particles could be seen by an electron microscope by negative staining with sodium phosphotungstate. The antibody titer gradually increased with the extension of the infection time within 49 days of the test [43]. Nawagitgul et al. applied the insect baculovirus expression system to express a recombinant Cap protein with a molecular weight of approximately 30 kDa. The test pigs were infected with PCV2, and the serum was detected using ImmunoSpot Technology. The antibodies against this recombinant protein indicated that the Cap encoded by the ORF2 gene is related to the immunoreactivity of PCV2 infection [8]. In short, ORF2 is currently the preferred target gene to prepare the recombinant vaccines for resisting the PCV2 virus [44,45,46].

Approximately 95% of animal body infections start in the mucous membranes (respiratory tract, gastrointestinal tract, reproductive tract, etc.), and mucosal immunization is an important protective barrier for the body. It offers several advantages over other routes of antigen delivery, including ease of administration, cost effectiveness, and the capacity of inducing both local and systemic immune responses [21,47,48,49]. *L. casei 393*, as the antigen delivery carrier to express PCV2 Cap, can adhere to and colonize the intestinal mucosa of a murine model and pigs and tolerate bile, thus serving as a promising candidate for the delivery of antigenic material to the mucosa in some vaccines [50,51]. Considering that most antigenic substances are not easily absorbed in the mucosa, to enhance the immune response effect, this study also introduced the LTB gene as a mucosal immune adjuvant.

To assess mucosal immune responses, specific IgA anti-Cap protein levels were examined from feces and vaginal wash. The oral administration of recombinant Cap-LTB-expressing *L. casei* could induce both systemic (IgG) and mucosal (IgA) immune responses. Specifically, IgA specific for Cap could be isolated from the gastrointestinal tract and vaginal wash compared to no detectable IgA anti-Cap responses in the control mice. The titers of anti-Cap IgG in the sera from mice immunized with pPG-2-Cap-LTB/*L. casei 393* were higher than those of mice immunized with pPG-1-Cap-LTB/*L. casei 393*, and both were higher than those of the control group, which indicated that secretory expression was more effective than surface-displayed expression to elicit the immune system responses. These results suggested that *L. casei* expressing recombinant Cap-LTB could be used in the vaccination of pigs, potentially protecting them from PCV2 infection; this vector successfully elicited significant and specific anti-Cap IgA and IgG responses, laying the foundation for the development of oral PCV vaccines.

## Figures and Tables

**Table 1:** Sequences of the primers.

Primer Names	Primer Sequences (5′-3′)
C0	GGATCC ^a^ AGGCATCTTCAACACCCGC
C1	ACTTTATTCATAACATACGGGCCCGGGCCGGGTTT ^b^ AAGTGGGGGGTC
L1	GACCCCCCACTTAAACCCGGCCCGGGCCCG ^b^ TATGTTATGAATAAAGT
L2	GGGCTCGAG ^a^ GTAGTTTTCCATACTGATTGCCGCA
A1	CAACTGCTGTCCCAGCTGTAG
A2	AGGAGGCGTTACCGCAGAAG
P1	TCTTTAAGATTAAATTCTCT
P2	ATGTAAACTACTCCTCCCGC

^a^ Restriction enzyme recognition sites used for cloning; ^b^ Flexible short peptides are shown in red.

## Data Availability

Not applicable.
